# Prevalence of risk factors among adolescents who attempted suicide: a cross-sectional study

**DOI:** 10.1590/1980-220X-REEUSP-2024-0197en

**Published:** 2024-11-04

**Authors:** Danton Matheus de Souza, Carlos Alberto dos Santos Treichel, Lucca Garcia Moreira Ribeiro, Ana Paula Scoleze Ferrer, Lisabelle Mariano Rossato

**Affiliations:** 1 Universidade de São Paulo, Escola de Enfermagem, Programa de Pós-Graduação em Enfermagem, São Paulo, SP, Brazil.; 2 Universidade de São Paulo, Escola de Enfermagem, Departamento de Enfermagem Materno Infantil e Psiquiátrica, São Paulo, SP, Brazil.; 3 Universidade de São Paulo, Escola de Enfermagem, São Paulo, SP, Brazil.; 4 Universidade de São Paulo, Faculdade de Medicina, São Paulo, SP, Brazil.

**Keywords:** Adolescent, Risk Factors, Suicide, Attempted, Adolescent Health, Mental Health, Emergency Medical Services, Adolescente, Factores de Riesgo, Intento de Suicidio, Salud del Adolescente, Salud Mental, Servicios Médicos de Urgencia

## Abstract

**Objective:**

To analyze the prevalence of risk factors among adolescents treated at an emergency department due to suicide attempt.

**Method:**

A cross-sectional, documentary, and retrospective study, conducted through the analysis of medical records of adolescents treated at an emergency department in a Brazilian teaching hospital from January 2015 to May 2023. Risk factors were divided into behavioral, health, violence, conflict, and stress. Data were subjected to descriptive and inferential analysis.

**Results:**

A total of 140 adolescent medical records were analyzed. Family conflicts (47.8%), previous suicide attempt (47.1%) and self-harm (30.7%) were the most prevalent risk factors. Risk factors were associated with age, sex, race, education, previous mental disorder and use of psychotropic medication (p < 0.05).

**Conclusion:**

A high prevalence of risk factors for suicide attempt in adolescents was observed, which was associated with sociodemographic characteristics. It is expected that this study will promote reflections on the translation of Brazilian public policies into care, especially in health promotion, with interventions that aim to reduce risk factors and enhance protective factors.

## RESUMEN

**Objetivo:**

Analizar la prevalencia de factores de riesgo entre adolescentes atendidos en un servicio de urgencias por intento de suicidio.

**Método:**

Estudio transversal, documental y retrospectivo, realizado a través del análisis de historias clínicas de adolescentes atendidos en un servicio de urgencia y emergencia de un hospital universitario brasileño, de enero de 2015 a mayo de 2023. Los factores de riesgo se dividieron en comportamentales, de salud, de violencia, conflicto y estrés. Los datos fueron sometidos a análisis descriptivo e inferencial.

**Resultados:**

Se analizaron 140 expedientes de adolescentes. Los conflictos familiares (47,8%), el intento previo de suicidio (47,1%) y la automutilación (30,7%) fueron los factores de riesgo más prevalentes. Los factores de riesgo se asociaron con edad, sexo, color, educación, trastorno mental previo y uso de medicación psicotrópica (p < 0,05).

**Conclusión:**

Se observó alta prevalencia de factores de riesgo para intento de suicidio en adolescentes, lo que se asoció con la caracterización sociodemográfica. Se espera que este estudio promueva reflexiones sobre la traducción de las políticas públicas brasileñas en asistencia, especialmente en promoción de la salud, con intervenciones que tengan como objetivo reducir los factores de riesgo y mejorar los factores protectores.

## INTRODUCTION

Mental disorders are a global public health problem that has been on the rise in recent years, especially among adolescents. This group represents approximately 16% of the global population and, due to the specificities of this stage of life, they experience numerous risk factors for mental health needs. It is estimated that more than 20% are likely to develop a mental disorder, with more than 50% of these cases beginning before the age of 14 and 75% before the age of 18. This reality directly impacts the lives of one in five adolescents^(1,2,3)^.

Adolescence is a crucial phase in human development with physical, psychological and social changes^(1)^. This is the beginning of the journey of building social identity, originality and self-image, in a search for autonomy, acceptance and occupation of collective spaces. The experiences of this phase shape the transition to adulthood, with long-term repercussions^(4,5)^. Social determinants, such as vulnerability and demands, can impact the process, and in order to face adversity, adolescents may engage in risky behaviors, such as suicidal behavior^(1)^.

Suicidal behavior consists of a series of actions that include ideation, planning, attempt and suicide^(6)^. The World Health Organization (WHO) estimates that, annually, more than 700 thousand people commit suicide, and for each death there are more than 20 suicide attempts, which leads to an estimate of more than 14 million attempts annually^(6)^. Furthermore, 77% of these occur in low and middle-income countries, such as Brazil, a place that is among the ten countries with the highest absolute numbers of suicides, with a growing increase^(7)^.

In Brazil, between 2011 and 2014, there were more than 67 thousand reports of suicides, with more than 15 thousand being among adolescents. Furthermore, in relation to suicide attempts, between 2007 and 2016, more than 12 thousand hospitalizations among adolescents were recorded due to the phenomenon^(8)^. These estimates may be underestimated, considering the low number of case reports^(6)^. However, the data are alarming and special attention should be paid to the phenomenon, especially in the prevention and health promotion process. To this end, it is essential to recognize risk factors.

In a descriptive and qualitative Brazilian study, it was observed that, when questioning 13 professionals from an interdisciplinary team about risk factors for suicide attempt in adolescents, they indicated that there is an attempt to draw attention, as they do not have emotional control, a low threshold for frustration and the family context does not act with reprisals^(5)^. This view reinforces the pathological idea of adolescence, in which behaviors are seen as problematic and deviant from a norm, making invisible not only suicidal behavior, but also mental health demands^(9)^.

In recent years, there has been an increase in research on risk factors for suicidal behavior. However, there is a focus on the adult population, and those that have worked with adolescents^(10,11,12)^ are restricted to highly developed countries. In Brazil, a study on risk factors was identified^(7)^, indicating female gender, mental disorders, use of alcohol and other drugs, changes in body image and family and social conflicts. However, this study was carried out with healthy adolescents, acting with the risk of ideation and planning. Thus, in order to advance the literature, it is necessary to identify the factors present in those who suicide attempt, considering the action resulting from exposure.

By understanding the factors that contribute to increased suicide risk among adolescents, child and adolescent mental healthcare professionals, educators and policy makers can develop targeted interventions that provide a safer and more supportive environment for these young people. In addition, research in this area contributes to changing mental health stigmas, promoting broader awareness and encouraging proactive actions to prevent suicide among adolescents. WHO, in its Live-Life guideline, highlights monitoring and assessment as a key pillar in reducing suicide attempts, providing a basis for guiding future prevention initiatives^(6)^. This study is in line with this principle by investigating the prevalence of risk factors in adolescents who have suicide attempt, contributing to the understanding and effective approach to these cases.

This study aims to analyze the prevalence of risk factors among adolescents treated at an emergency department for suicide attempt.

## METHOD

### Study Design

This is an observational, cross-sectional, documentary and retrospective study. To guide the writing of this study, the STrengthening the Reporting of OBservational studies in Epidemiology (STROBE) for cross-sectional studies was used^(13)^.

### Location, Population and Selection Criteria

Data were collected through analysis of medical records of adolescents treated for suicide attempt in an emergency department. It is worth reiterating that the choice of collection location is justified by the estimate that more than a third of adolescents who attempt suicide are initially treated in emergency rooms^(5,8)^. The department is part of the structure of a public teaching hospital located in the city of São Paulo, Brazil, which houses the Children’s Emergency Room (CER), responsible for providing care to adolescents aged between 10 and 15 years old, and the Adult Emergency Room (AER), which serves individuals aged between 15 and 19 years old.

The study was conducted between August and November 2023. Based on the WHO classification, all adolescents, defined as those aged between 10 and 19 years old^(1)^, who received care due to suicide attempt in the services analyzed from January 2015 to May 2023 were considered eligible for the study. Taking into account previous notes in the literature on the percentage of incorrect classifications and underreporting of suicide attempt cases^(4,8)^, to identify these, in addition to care related to codes X60 to X84 (suicide attempt) of the International Statistical Classification of Diseases and Related Health Problems, 10^th^ revision (ICD-10), codes T36 to T78 (poisonings), X60 to X84 (intentional self-harm), T50.9 to T65.9 (exogenous intoxications of undetermined intent) and Y09 to X34 (events of undetermined intent) were considered. No exclusion criteria were established.

### Data Collection

Through the Medical Archive and Statistics Service (SAME – *Serviço de Arquivo* Médico e *Estatística*) of the studied services, 420 medical records related to the above-mentioned codes were located. All of these records were obtained in full and assessed, and cases in which there was documentation of intentionality were characterized as suicide attempts, evidencing, implicitly or explicitly, adolescents’ desire to take their own lives^(6)^. Among the medical records analyzed, 140 characterized suicide attempts and were included in the study. Medical record assessment and subsequent data extraction were conducted by a nurse, a specialist in child and adolescent health, with clinical experience in treating adolescents who suicide attempt.

To extract the data, a form developed by the researchers was used, consisting of objective questions, with closed-ended and open-ended answer options. Data were collected regarding adolescent sociodemographic characteristics: sex; color (white and non-white (brown, black, indigenous and yellow)); age; and education (up to elementary school (ES), high school (HS) and higher education (HE)). Data were also collected on previous diagnosis of mental disorders, current use of psychotropic medications and behavioral risk factors: previous suicide attempt; self-harm; being LGBTQIA+; previous and/or current use of psychoactive substances (PAS); history of school dropout and poor academic performance; health factors: report of grief the death of a loved one; history of previous psychiatric hospitalization; changes in the perception of body image; lack of timely support in the Psychosocial Care Network (RAPS – *Rede de Atenção Psicossocial*); and report of victimization by bullying; and the factors of violence, conflict and stress: history of victimization by physical, psychological and sexual violence; report of family conflict and love; separation of parents; and history of suicide attempt by a loved one. These are the independent variables. It is reiterated that the risk factors were determined from previous studies^(10,12,14,15)^.

To characterize the previous diagnosis of mental disorders, the record of any diagnosis related to section F of ICD-10 was considered. Current use of psychotropic drugs was considered in cases where there was a record of regular use of some psychotropic medication, belonging to group N of the anatomical, therapeutic and chemical (ATC) classification of the WHO in the 30 days prior to care^(16)^.

### Data Analysis and Treatment

The data were tabulated and submitted to descriptive and inferential analysis using Stata18^®^ (Stata Corporation, College Station, Texas, USA). For descriptive analysis, percentiles, measures of central tendency (mean) and dispersion (standard deviation) were used, and for inferential analysis, after testing the nature of the variable’s distribution, Pearson’s chi-square test was used to verify the existence of an association between the dependent and independent variables. An alpha of 0.05 (or 5%) and a 95% Confidence Interval were considered to minimize the occurrence of type II errors.

### Ethical Aspects

The study received ethical approval from the *Universidade de São Paulo* School of Nursing Research Ethics Committee, under Opinion 6,128,602, June 19, 2023, and from the co-participating institution Research Ethics Committee, under Opinion 6,182,807, July 14, 2023. The ethical guidelines established in Resolutions 466/12 and 510/16 of the Brazilian National Health Council were respected. The signing of the Informed Consent Form was waived, with the signing of the Term of Responsibility by the main researcher.

## RESULTS

Data were collected from 140 medical records of adolescents treated for suicide attempt. The mean age was 16 years (SD: 2.2). The majority were female (80.7%), white (80.7%) and had completed high school (54.3%). A previous diagnosis of mental disorders was found in 52.1% of cases, with 36.4% of adolescents using psychotropic medications. Regarding risk factors, the high prevalence of family conflicts (47.8%), previous suicide attempt (47.1%) and self-harm (30.7%) stood out (Table 1).

**Table 1:** Participant and risk factor characterization (N* = 140) – São Paulo, SP, Brazil, 2023.

Participant characterization		
Variables	Mean (+SD*)	Min-Max
**Age (years)**	16.3 (+2.2)	10–19
	N*	**Prevalence (%)**
**Sex**		
Female	113	80.7%
Male	27	19.3%
**Color**		
White	113	80.7%
Non-white	27	19.3%
**Age**		
10 to 14 incomplete years	35	25.0%
15 to 19 incomplete years	105	75.0%
**Education**		
Up to ES^‡^	44	31.4%
Up to HS^§^	76	54.3%
Up to HE^||^	20	14.3%
**Mental disorders**	73	52.1%
**Current use of psychotropic medications**	51	36.4%
**Risk factors**		
	N*	**Prevalence (%)**
**Behavioral risk factors**		
Previous suicide attempt	66	47.1%
Self-harm	43	30.7%
LGBTQIA^+¶^	13	9.3%
Previous or current use of PAS**	21	15.0%
History of dropping out of school	4	2.8%
Poor academic performance	19	13.8%
**Health risk factors**		
Grief	6	4.3%
Previous psychiatric hospitalization	2	1.4%
Changes in body image perception	8	5.7%
Lack of support in RAPS^††^	1	0.7%
Bullying	15	10.7%
**Violence, conflict and stress risk factors**		
Physical violence	5	3.8%
Psychological violence	21	15.0%
Sexual violence	10	7.1%
Family conflict	67	47.8%
Love conflict	24	17.1%
Divorced parents	16	11.4%
SA^‡‡^ in someone close	5	3.6%

Note: *N = number of adolescents;

^†^SD = standard deviation;

^‡^ES = elementary school;

^§^HS = high school;

^||^HE = higher education;

^¶^LGBTQIA+ = lesbian, gay, bisexual, transgender, queer, intersex, asexual and others;

**PAS = psychoactive substances;

^††^RAPS = Psychosocial Care Network;

^‡‡^SA = suicide attempt.

From the inferential analysis, it was observed that the age of 10–14 years was associated with victimization by bullying (p = 0.007), physical violence (p = 0.004) and psychological violence (p = 0.002), report of family conflict (p < 0.001) and divorced parents (p = 0.002). Males was associated with the use of PAS (p = 0.01) and grief (p = 0.05). Being a non-white adolescent was associated with a history of school dropout (p < 0.001) and sexual violence (p = 0.01). Those who studied up to ES showed an association with the report of family conflicts (p = 0.001) and suicide attempt by a loved one (p = 0.05). Among adolescents who studied up to HS, an association was observed with lack of support in RAPS (p = 0.002). Previous diagnosis of mental disorders was associated with previous suicide attempt (p < 0.001), being LGBTQIA+ (p = 0.01), changes in body image perception (p = 0.005) and lack of support in RAPS (p = 0.002). Current use of psychotropic medications was associated with a history of previous suicide attempts (p = 0.005), being LGBTQIA+ (p < 0.05) and the presence of changes in body image perception (p = 0.02). Tables 2, 3 and 4 present the results of the association tests between dependent variables and each of the risk factors included in the study.

**Table 2:** Association between characterization variables and behavioral risk factors (N* = 140) – São Paulo, SP, Brazil, 2023.

	Previous SA^†^	p^‡^	Self-mutilation	p^‡^	LGBTQIA+^§^	p^‡^	Use of PAS^||^	p^‡^	School dropout	p^‡^	Low academic performance	p^‡^
**Sex**												
Female	52 (46.0%)	0.585	37 (32.7%)	0.287	9 (7.9%)	0.271	13 (11.5%)	**0.018**	2 (1.7%)	0.114	14 (12.4%)	0.403
Male	14 (51.8%)		6 (22.2%)		4 (14.8%)		8 (29.6%)	2 (7.4%)	5 (18.5%)			
**Color**												
White	50 (44.2%)	0.160	36 (31.8%)	0.548	12 (10.6%)	0.266	14 (12.4%)	0.077	0 (0)	**<0.001**	17 (15.0%)	0.298
Non-white	16 (59.3%)		7 (25.9%)		1 (3.7%)		7 (25.9%)		4 (14.8%)		2 (7.4%)	
**Age**												
10–14 years	12 (34.3%)	0.078	14 (40.0%)	0.169	2 (5.7%)	0.401	4 (11.4%)	0.494	2 (5.7%)	0.241	5 (14.3%)	0.887
15–19 years	54 (51.4%)		29 (27.2%)		11 (10.5%)		17 (16.2%)		2 (1.9%)		14 (13.3%)	
**Education**												
Up to ES^¶^	15 (34.1%)	0.106	15 (35.1%)	0.839	3 (6.8%)	0.791	7 (15.9%)	0.795	2 (4.5%)	0.590	7 (15.9%)	0.160
Up to HS**	41 (53.9%)		22 (28.9%)		8 (10.5%)		12 (15.8%)		2 (2.6%)		7 (9.2%)	
Up to HE^††^	10 (50.0%)		6 (30.0%)		2 (10.0%)		2 (10.0%)		0 (0)		5 (25.0%)	
**Mental disorders**												
Yes	45 (61.6%)	**<0.001**	25 (34.2%)	0.344	11 (15.1%)	**0.014**	11 (15.1%)	0.981	3 (4.1%)	0.353	11 (15.1%)	0.589
No	21 (31.3%)		18 (26.8%)		2 (2.9%)		10 (14.9%)		1 (1.5%)		8 (11.9%)	
**Continuous medication**												
Yes	32 (62.7%)	**0.005**	20 (39.2%)	0.099	8 (15.7%)	**0.048**	7 (13.7%)	0.749	3 (5.8%)	0.104	9 (17.6%)	0.286
No	34 (38.2%)		23 (25.8%)		5 (5.6%)		14 (15.7%)		1 (1.1%)		10 (11.2%)	

Note: *N = number of adolescents;

^†^SA = suicide attempt;

^‡^p = p-value;

^§^LGBTQIA+ = lesbians, gays, bisexuals, transgender, queers, intersex, asexual and others;

^||^PAS = psychoactive substances;

^¶^ES = elementary school;

**HS = high school;

^††^HE = higher education.

**Table 3:** Association between characterization variables and health risk factors (N* = 140) – São Paulo, SP, Brazil, 2023.

	Grief	p^†^	Previous psychiatric hospitalization	p^†^	Change in body image	p^†^	Lack of welcoming	p^†^	Bullying	p^†^
**Sex**										
Female	3 (2.6%)	**0.05**	1 (0.8%)	0.267	7 (6.2%)	0.616	1 (0.8%)	0.667	14(12.4%)	0.190
Male	3 (11.1%)		1 (3.7%)		1 (3.7%)		0 (0)		1 (3.7%)	
**Color**										
White	5 (4.4%)	0.868	1 (0.8%)	0.267	7 (6.2%)	0.616	1 (0.8%)	0.667	14 (12.4%)	0.190
Non-white	1 (3.7%)		1 (3.7%)		1 (3.7%)		0 (0)		1 (3.7%)	
**Age**										
10–14 years	2 (5.7%)	0.630	0 (0)	0.411	3 (8.6%)	0.4	0 (0)	0.837	8 (22.8%)	**0.007**
15–19 years	4 (3.8%)		2 (1.9%)		5 (4.7%)		1 (0.9%)		7 (6.8%)	
**Education**										
Up to ES^‡^	3 (6.8%)	0.543	1 (2.3%)	0.771	1 (2.8%)	0.416	0 (0)	**0.002**	7 (15.9%)	0.162
Up to HS^§^	2 (2.6%)		1 (1.3%)		5 (6.9%)		1 (1.3%)		8 (10.5%)	
Up to HE^||^	1 (5.0%)		0 (0)		2 (10.0%)		0 (0)		0 (0)	
**Mental disorders**										
Yes	2 (2.7%)	0.346	1 (1.4%)	0.951	8 (10.9%)	**0.005**	1 (1.4%)	**0.002**	6 (8.2%)	0.319
No	4 (5.9%)		1 (1.5%)		0 (0)		0 (0)		9 (13.4%)	
**Continuous medication**										
Yes	1 (1.9%)	0.304	1 (1.9%)	0.688	6 (11.7%)	**0.02**	0 (0)	0.098	5 (9.8%)	0.792
No	5 (5.6%)		1 (1.2%)		2 (2.2%)		1 (1.1%)		10 (11.2%)	

Note: *N = number of adolescents;

^†^p = p-value;

^‡^ES = elementary school;

^§^HS = high school;

^||^HE = higher education.

**Table 4:** Association between characterization variables and factors of violence, conflict and stress (N* = 140) – São Paulo, SP, Brazil, 2023.

	Physical violence	p*	Psychological violence	p*	Sexual violence	p*	Family conflict	p*	Love conflict	p*	Divorced parents	p*	SA^†^ close	p*
**Sex**														
Female	4 (3.5%)	0.967	19 (16.8%)	0.219	7 (6.2%)	0.373	54 (47.8%)	0.973	19(16.8%)	0.833	14 (12.4%)	0.465	4 (3.5%)	0.967
Male	1 (3.7%)		2 (7.4%)		3 (11.1%)		13 (48.1%)		5 (18.5%)		2 (7.4%)		1 (3.7%)	
**Color**														
White	4 (3.5%)	0.967	15 (13.3%)	0.242	5 (4.4%)	**0.011**	55 (48.7%)	0.693	20 (17.7%)	0.721	13 (11.5%)	0.954	4 (3.5%)	0.967
Non-white	1 (3.7%)		6 (22.2%)		5 (18.5%)		12 (44.4%)		4 (14.8%)		3 (11.1%)		1 (3.7%)	
**Age**														
10–14 years	4 (11.4%)	**0.004**	11 (31.4%)	**0.002**	4 (11.4%)	0.256	27 (77.1%)	**<0.001**	3 (8.6%)	0.12	9 (25.7%)	**0.002**	2 (5.7%)	0.430
15–19 years	1 (0.9%)		10 (9.5%)		6 (5.7%)		40 (38.1%)		21 (20.0%)		7 (6.6%)		3 (2.8%)	
**Education**														
Up to ES^‡^	3 (6.8%)	0.319	6 (13.6%)	0.085	4 (9.1%)	0.808	27 (61.3%)	**0.001**	8 (18.2%)	0.952	9 (20.4%)	0.071	4 (9.1%)	**0.05**
Up to HS^§^	2 (2.6%)		15 (19.7%)		5 (6.6%)		38 (50.0%)		13 (17.1%)		6 (7.8%)		1 (1.3%)	
Up to HE^||^	0 (0)		0 (0)		1 (5.0%)		2 (10.0%)		3 (15.0%)		1 (5.0%)		0 (0)	
**Mental disorders**														
Yes	2 (2.7%)	0.580	12 (16.4%)	0.619	5 (6.8%)	0.888	35 (47.9%)	0.983	10 (13.7%)	0.259	9 (12.3%)	0.727	3 (4.1%)	0.720
No	3 (4.5%)		9 (13.4%)		5 (7.4%)		32 (47.7%)		14 (20.9%)		7 (10.4%)		2 (2.9%)	
**Continuous medication**														
Yes	2 (3.9%)	0.866	9 (17.6%)	0.507	5 (9.8%)	0.355	24 (47.1%)	0.886	6 (11.7%)	0.201	8 (15.7%)	0.231	3 (5.8%)	0.265
No	3 (3.4%)		12 (13.5%)		5 (5.6%)		43 (48.3%)		18 (20.2%)		8 (8.9%)		2 (2.2%)	

Note: *N = number of adolescents;

*p = p-value;

^†^SA = suicide attempt;

^‡^ES = elementary school;

^§^HS = high school;

^||^HE = higher education.

## DISCUSSION

The literature^(6,10,17)^ indicates that the end of adolescence is the most critical stage of development, due to the proximity of the transition to adulthood, increasing demands, responsibilities, family and social expectations. Adolescents between 15 and 19 years of age tend to attempt suicide four times more than those between 10 and 14 years of age^(6)^. This data supports the findings of this study, with the prevalence of adolescents between 15 and 19 years of age. However, special attention should be paid to the entire phase of adolescence.

In this study, 52.1% of adolescents had a mental disorder, associated with numerous risk factors. This finding supports the literature^(6,9)^, which also indicates that mental disorders increase the possibility of suicide attempt by three times^(17)^. There is an emerging need to look at RAPS, with progress in translating public policies into clinical practice, and strengthening the service, especially in its promotion, early identification and monitoring actions, with community-based actions, which move away from the hospital-centric and biomedical vision, which guided mental healthcare for years and which still influence child and adolescent care practice^(9)^. Among the associations in this study, one that deserves attention is the previous suicide attempt, which was also associated with the use of continuous psychotropic medications.

The literature indicates that one in four people who commit suicide had already suicide attempt at least once^(18)^. This supports a retrospective cohort study of 25,037 young people aged 3 to 25 years who were treated at a healthcare service for mental health needs. In this study, it was observed that 1,766 adolescents had suicide attempt in the last six months, this factor being the strongest predictor of a new attempt, with a rate four times higher^(19)^. A meta-analysis demonstrated that the risk of repetition is associated with females, due to less violent attempts, when compared to men^(12)^, although this study did not demonstrate this association.

In recurrent suicide attempts, hospitalization tends to be the conduct, however it is important to reflect on this context. In this study, previous psychiatric hospitalization was not associated with any dependent variable; in the literature, it is observed that suicide is more frequent in this group, especially in the three months after discharge^(18)^. In Brazil, after the consolidation of the Psychiatric Reform, the number of hospitalizations due to mental health problems decreased, with the transition from hospital-centered care to territorial-based care, with emphasis on the Psychosocial Care Center. Despite the potential of this apparatus in RAPS, many professionals still tend to act with mental healthcare medicalization, as seen in this study, however it is worth noting that hospital-centric practices and mental healthcare medicalization are questioned in the literature, as, in isolation, they do not promote potential effects^(6,12,18)^.

In the process of caring for mental health, the family can be a catalyst for improvement, as well as promote new problems. This was seen in a cross-sectional study with 22,126 Mexican adolescents, in which the presence of parents at home, a good relationship and a pleasant atmosphere were protective factors against suicide attempt. In cases where the family atmosphere was classified as bad, the chance of a suicide attempt increased more than twofold, and when there was a conflict, the chance increased threefold^(17,19)^. Another cross-sectional study, conducted with 246 adolescents in Colombia, showed that 66% presented some type of family dysfunction, with mental disorders being a predictor^(2)^. This finding supports this study, with family conflict being the most prevalent risk factor, which can be caused by criticism, demands, broken expectations and difficulty in communication. Conflicts can be even greater in cases where adolescents drop out of school, as seen here, with an association between conflicts and adolescents who studied up to ES.

In adverse situations during adolescence, grief for loved ones may occur, as seen in 4.3% of the sample in this study, associated with sexuality, and this supports a review of previous literature, which demonstrated that complicated grief may lead to the risk of suicidal behavior and other mental disorders^(20)^. In the case of adolescents of sexuality, this may occur due to their social representation, constructed since childhood, in which the expression of emotions must be repressed to fit into an ideal pattern.

Another occurrence is suicide attempt and/or suicide in family members. The literature indicates that the prevalence of mental disorders is above 90% in family members of individuals who have committed suicide^(13)^, which can also lead to suicidal behavior in family members, especially younger ones^(21)^. This scenario can translate into the loss of loved ones, such as friends. Here, this factor was associated with adolescents who studied up to ES. Family relationships can be impacted by numerous factors, which leads adolescents to seek support and acceptance in other social contexts, such as school.

Bullying is common in schools, as seen in a review that indicated estimates between 18% and 31%. Furthermore, this study demonstrated that experiencing the phenomenon leads to social isolation, hopelessness, sadness, lack of social skills and impulsivity. Other impacts include poor performance and subsequent school dropout^(10)^, factors seen in this study. To reframe this experience, it is necessary for adolescents to acquire coping skills, which may not occur at the beginning of this new phase of life, a hypothesis for the association between bullying and adolescents between 10 and 14 years old, as seen in this study.

It is worth noting that some of the risk factors can initially be experienced in schools, which demonstrates the need for coordination and strengthening of this care network. In Brazil, in January 2024, the Brazilian National Policy for Psychosocial Care in School Communities was approved, which advances school action, ensuring the promotion of mental health in this location, with the integration of different territorial services, guaranteeing psychosocial care for children and adolescents at risk^(22)^.

Hence, exposure to violence was associated with age and race, with psychological violence being the most prevalent. The literature indicates that victimization of violence leads to risk behaviors, such as use of PAS, changes in body image perception, self-harm and other mental disorders, being more common in children under 16 years old^(23)^, supporting this study that demonstrated an association between violence and adolescents between 10 and 14 years old. Cross-sectional research, conducted with young people between 13 and 24 years old, in Zambia (Africa), showed that 76.8% were victims of at least one act of violence; of these, 42.4% reported mental distress in the last 30 days and, 12.5%, suicidal behavior^(24)^. The data is alarming, and it is necessary to reflect on strategies to give visibility to violence, which is trivialized within common sense and made invisible in RAPS.

Another risk factor for suicide attempt is being part of the LGBTQIA+ community, a group exposed to prejudice due to historical conservatism, which provides exposure to numerous risk factors for suicidal behavior. A population survey conducted in the United States with 2,209 LGBTQIA+ individuals between the ages of 12 and 29 who committed suicide showed a higher prevalence in transgender men and bisexual women, a higher probability of recurrent suicide attempts, mental disorders and use of medications^(25)^, similar to this study. Special attention should be paid to recurrent attempts, as seen in a study in which, of 13,852 LGBTQIA+ adolescents, 276 suicide attempt once, 116, between 2–3 times, 37, between 4–9 times, 19, more than ten times, and 22, more than ten times^(26)^.

In Brazil, a population-based study conducted with 510 adolescent HS students indicated a prevalence of suicide risk of 17.3%, a high number, and this risk was 73% higher in those with changes in body perception^(7)^. Changes in body image are a public health problem, but they are still largely ignored, especially in the adult population, when their repercussions are more pronounced and lead to chronic diseases and/or imminent risk to life. In South Korea, a cross-sectional study demonstrated an association between changes in body image, both obesity and malnutrition, and attempted and/or completed suicide^(27)^, which supports this study, this risk factor being associated with mental health problems and continuous use of psychotropic drugs.

In the midst of imminent mental distress, adolescents may use PAS as a way to escape reality. In adults, it has been established that PAS use can alleviate undesirable symptoms, however, when it becomes the only form of recreation, the risk of suicide attempts increases^(11)^. In adolescents, the use of PAS is a predictor of already established suicidal behavior^(7,17)^, which supports this study. Furthermore, here the use of PAS was associated with males, an association already established in the literature^(7,11,17)^.

Although self-harm was not associated with the variables of this study, its prevalence was high (30.7%), supporting the literature^(14,18)^. In meta-synthesis, it was observed that young people reported self-harm as a way of expressing their emotions, with relief from emotional pain, compensated by physical pain^(14)^. Furthermore, it is worth noting that this behavior, despite being a risk factor for suicide attempt, may not be associated with a desire to die and should be investigated in a stigma-free care setting^(18)^.

Furthermore, it is necessary to look at other health determinants, such as income, housing, food security, social support, among others, which intersect, increasing risk factors and maintaining cycles of mental distress^(6,7)^. In this context, skin color deserves attention, such as black adolescents, a group that historically has unequal access to social rights, and when they do access them, they experience oppression, increasing vulnerability^(28)^. Here, non-white adolescents were associated with school dropout and sexual violence.

In this study, only one adolescent had documentation of the lack of care in RAPS. Despite the low prevalence, it is known that this phenomenon is more frequent. Some points may be associated with the lack of timely care, such as: the reduced number of public policies for adolescent health, with those that exist focusing on the prevention of PAS use, sexual and reproductive health; the loss of connection between adolescents and RAPS, as in primary care, where childcare consultations focus on early childhood to the detriment of other phases of human development; and the prior search for the service, with hostility by the professional, in a service marked by stigmas, strengthening the view that the service is not a potential place for their life^(3,4,7,14)^.

There is a lack of spaces for adolescents in contemporary times, and acting on risk factors intrinsically demands a redefinition of this context, with the reduction of the marginalization of this public before RAPS, which must be operating in an interconnected manner^(9)^. Progress has been made in recent years, with the Brazilian Psychiatric Reform, the implementation of territorially based services and new intersectoral health policies^(12,18,22)^. Even so, there is still a long way to go and, to this end, we highlight the essential role of implementation research in translating public policies into clinical practice, especially in places where access to them is not yet guaranteed.

In care organization in RAPS, primary and specialized care deserve attention. In adults, a Brazilian study shows that the interconnection of these devices is almost non-existent, and, in attempts at articulation, a loss is noted in the network, with only one service being held responsible to the detriment of the others^(29)^. This interconnection can be more challenging in the case of adolescents, due to the aspects mentioned above in this discussion.

Although this study focuses on isolated risk factors, as demonstrated throughout this line of argument, there is a sum of factors that leads to intensification of vulnerabilities and distress. It is worth reflecting on strategies to change this scenario, with primary care, as it is close to adolescents in their territory, being an essential tool of RAPS. It is recommended to establish awareness practices on mental health, dealing with stigma, and early detection of suicidal behavior, looking at risk factors interconnected with social determinants, from an intersectoral, territorial and interdisciplinary perspective, aiming to strengthen protective factors^(6,10,18,30)^. Identifying the risk factors mentioned here and acting to modify them is the basis of the line of care that aims to modify this scenario.

In this study, there was a dependence on documentation from healthcare professionals on risk factors and social determinants, which leads to a limitation, since prevalence estimates may be over- or underestimated. However, the data presented are potential and innovative for expanding the frontier of international knowledge on suicidal behavior, contributing to nursing and literature.

Given the contributions of the study to the area of nursing, the possibility of qualifying care for adolescents in RAPS is highlighted, with attention to those who have risk factors for suicidal behavior, allowing rapid interventions that can reduce its high prevalence. It is important to emphasize that nursing actions in the face of this phenomenon should not only occur in specialized mental healthcare services, but rather throughout RAPS, with this study being an opening for professionals to approach and empower themselves with risk factors, with theoretical knowledge that should guide actions in clinical practice. In the scientific literature, the contribution of this study is indicated with new estimates of prevalence of risk factors in a Brazilian scenario, which should be worked on in future studies that aim to modify this scenario, promoting mental health and reducing recurrences of suicide attempts.

## CONCLUSION

This study demonstrated the prevalence of behavioral, health, violence, conflict and stress risk factors, with family conflicts (47.8%), previous suicide attempt (47.1%) and self-harm (30.7%) being the most prevalent. Risk factors were associated with age, sex, race, education, previous mental disorder and use of psychotropic medication.
