# ERRATUM: Prevalence of risk factors among adolescents who suicide attempt: a cross-sectional study

**DOI:** 10.1590/1980-220X-REEUSP-2024-0197eren

**Published:** 2025-01-27

In the article “Prevalence of risk factors among adolescents who suicide attempt: a cross-sectional study”, with DOI number: https://doi.org/10.1590/1980-220X-REEUSP-2024-0197en, published in the Revista da Escola de Enfermagem da USP, volume 58:e20240197 of 2024, p.1–10, on page 1:

**Where it was written:**

Prevalence of risk factors among adolescents who suicide attempt: a cross-sectional study

**Now read:**

Prevalence of risk factors among adolescents who attempted suicide: a cross-sectional study

