# Supplementary material for: Prevalence of risk factors among adolescents who attempted suicide: a cross-sectional study
Source: Rev Esc Enferm USP. 2024 Nov 4;58:e20240197. doi: 10.1590/1980-220X-REEUSP-2024-0197en (PMC11552609; doi:10.1590/1980-220X-REEUSP-2024-0197en)
Supplement: Translation

# Translation: Prevalência de fatores de risco entre adolescentes que tentaram suicídio: estudo transversal

**DOI:** 10.1590/1980-220X-REEUSP-2024-0197pt

**Authors:** Danton Matheus de Souza, Carlos Alberto dos Santos Treichel, Lucca Garcia Moreira Ribeiro, Ana Paula Scoleze Ferrer, Lisabelle Mariano Rossato

**Affiliations:** 1 Universidade de São Paulo, Escola de Enfermagem, Programa de Pós-Graduação em Enfermagem, São Paulo, SP, Brasil.; 2 Universidade de São Paulo, Escola de Enfermagem, Departamento de Enfermagem Materno Infantil e Psiquiátrica, São Paulo, SP, Brasil.; 3 Universidade de São Paulo, Escola de Enfermagem, São Paulo, SP, Brasil.; 4 Universidade de São Paulo, Faculdade de Medicina, São Paulo, SP, Brasil.

**Keywords:** Adolescente, Fatores de Risco, Tentativa de Suicídio, Saúde do Adolescente, Saúde Mental, Serviços Médicos de Emergência

## Abstract

**Objetivo:**

Analisar a prevalência de fatores de risco entre adolescentes atendidos em um departamento de urgência e emergência por tentativa de suicídio.

**Método:**

Estudo transversal, documental e retrospectivo, conduzido por meio da análise de prontuários de adolescentes atendidos em um departamento de urgência e emergência em um hospital escola brasileiro de janeiro de 2015 a maio de 2023. Os fatores de risco foram divididos em comportamentais, de saúde, de violência, de conflito e de estresse. Os dados foram submetidos à análise descritiva e inferencial.

**Resultados:**

Foram analisados 140 prontuários de adolescentes. Os conflitos familiares (47,8%), tentativa de suicídio prévia (47,1%) e automutilação (30,7%) foram os fatores de risco mais prevalentes. Os fatores de risco se associaram à idade, sexo, cor, escolaridade, transtorno mental prévio e uso de medicação psicotrópica (p < 0,05).

**Conclusão:**

Observou-se uma alta prevalência de fatores de risco à tentativa de suicídio em adolescentes que se associou à caracterização sociodemográfica. Espera-se que este estudo promova reflexões sobre a tradução das políticas públicas brasileiras à assistência, em especial na promoção da saúde, com intervenções que visem reduzir os fatores de risco e potencializar os de proteção.

## INTRODUÇÃO

Os transtornos mentais são um problema de saúde pública mundial, com aumento crescente nos últimos anos, especialmente em adolescentes. Esse público representa, aproximadamente, 16% da população global e, pelas especificidades dessa fase da vida, vivenciam inúmeros fatores de risco às demandas de saúde mental. Estima-se que mais de 20% estão propensos a desenvolver um transtorno mental, sendo que mais de 50% desses casos começam antes dos 14 anos e 75% antes dos 18 anos. Essa realidade impacta diretamente a vida de um em cada cinco adolescentes^(1,2,3)^.

A adolescência é uma fase crucial no desenvolvimento humano com mudanças físicas, psicológicas e sociais^(1)^. Há o início da trajetória da construção da identidade social, originalidade e autoimagem, em uma busca por autonomia, aceitação e ocupação de espaços coletivos. As experiências dessa fase moldam a transição à vida adulta, com repercussões a longo prazo^(4,5)^. Os determinantes sociais, como as vulnerabilidade e exigências, podem impactar o processo, e, de forma a enfrentar as adversidades, os adolescentes podem aderir a comportamentos de risco, tal como o comportamento suicida^(1)^.

O comportamento suicida consiste em uma série de ações que incluem a ideação, o planejamento, a tentativa e o suicídio^(6)^. A Organização Mundial da Saúde (OMS) estima que, anualmente, mais de 700 mil pessoas se suicidam, e a cada óbito há mais de 20 tentativas de suicídio, o que leva a uma estimativa de mais de 14 milhões de tentativas anuais^(6)^. Ainda, 77% dessas ocorrem em países de baixa e média renda, como o Brasil, local que integra os dez países com maiores números absolutos de suicídio, com aumento crescente^(7)^.

No Brasil, entre 2011 e 2014, houve mais de 67 mil notificações de suicídios, com mais de 15 mil sendo de adolescentes. Ainda, em relação às tentativas de suicídio, entre 2007 e 2016, foram registradas mais de 12 mil hospitalizações em adolescentes devido ao fenômeno^(8)^. Essas estimativas podem estar subestimadas, considerando o baixo número de notificações dos casos^(6)^. Todavia, os dados são alarmantes e atenção especial deve se voltar ao fenômeno, especialmente no percurso de prevenção e promoção da saúde. Para tal, é essencial reconhecer os fatores de risco.

Em estudo descritivo e qualitativo brasileiro, observouse que, ao questionar 13 profissionais de uma equipe interdisciplinar sobre os fatores de risco à tentativa de suicídio em adolescentes, esses indicaram que há uma tentativa de chamar atenção, por não possuírem controle emocional, baixo limiar à frustração e o contexto familiar não atuar com represálias^(5)^. Essa visão reforça a ideia patológica da adolescência, em que os comportamentos são vistos como problemáticos e desviantes de uma norma, invisibilizando não só o comportamento suicida, mas também as demandas de saúde mental^(9)^.

Nos últimos anos, houve um aumento de investigações sobre os fatores de risco ao comportamento suicida. Contudo, há enfoque na população adulta, e aqueles que atuaram com adolescentes^(10,11,12)^ se restringem a países de alto desenvolvimento. No Brasil, identificou-se estudo sobre os fatores de risco^(7)^, sendo indicados o sexo feminino, transtornos mentais, uso de álcool e outras drogas, alterações na imagem corporal e conflitos familiares e sociais. Porém, esse estudo foi realizado em adolescentes hígidos, atuando com o risco da ideação e planejamento. Assim, para o avançar na literatura, é necessário identificar os fatores presentes naqueles que tentaram suicídio, considerando a ação decorrente da exposição.

Ao compreender os elementos que contribuem para o aumento do risco de suicídio entre os adolescentes, os profissionais de saúde mental e infantojuvenil, educadores e formuladores de políticas podem desenvolver intervenções direcionadas, proporcionando um ambiente mais seguro e de apoio para esses jovens. Além disso, a pesquisa nessa área contribui para alterar os estigmas da saúde mental, promovendo uma conscientização mais ampla e encorajando ações proativas para prevenir o suicídio entre os adolescentes. A OMS, em seu *guideline Live-Life*, destaca a monitorização e a avaliação como um pilar fundamental na redução de tentativas de suicídio, proporcionando uma base para orientar iniciativas de prevenção futuras^(6)^. Este estudo se alinha a esse princípio, ao investigar a prevalência de fatores de risco em adolescentes que já tentaram suicídio, contribuindo para a compreensão e abordagem eficaz dos casos.

Este estudo objetiva analisar a prevalência de fatores de risco entre adolescentes atendidos em um departamento de urgência e emergência por tentativa de suicídio.

## MÉTODO

### Desenho do Estudo

Estudo observacional, do tipo transversal, documental e retrospectivo. Para guiar a redação deste estudo, utilizou-se o instrumento *STrengthening the Reporting of OBservational studies in Epidemiology* (STROBE) para estudos transversais^(13)^.

### Local, População e Critérios de Seleção

Os dados foram coletados por meio da análise de prontuários de adolescentes atendidos devido à tentativa de suicídio em um departamento de urgência e emergência. Reitera-se que a escolha do local de coleta se justifica pela estimativa de que mais de um terço dos adolescentes com tentativa de suicídio é, inicialmente, atendido em prontos-socorros^(5,8)^. O departamento integra a estrutura de um hospital escola público localizado no município de São Paulo, Brasil, que abriga o Pronto-Socorro Infantil (PSI), responsável por prestar atendimento a adolescentes com idade entre 10 e 15 anos incompletos, e o Pronto-Socorro Adulto (PSA), que atende indivíduos com idade entre 15 e 19 anos incompletos.

O estudo foi conduzido entre agosto e novembro de 2023. Com base na classificação da OMS, foram considerados elegíveis para o estudo todos os adolescentes, definidos como aqueles com idades entre 10 e 19 anos incompletos^(1)^, que receberam atendimento devido à tentativa de suicídio nos serviços analisados durante o período de janeiro de 2015 a maio de 2023. Levando em conta apontamentos prévios na literatura sobre o percentual de classificações incorretas e a subnotificação dos casos de tentativa de suicídio^(4,8)^, para identificação desses, além dos atendimentos relacionados aos códigos X60 a X84 (tentativa de suicídio) da Classificação Estatística Internacional de Doenças e Problemas Relacionados à Saúde, 10° edição (CID-10), foram considerados os códigos T36 a T78 (envenenamentos), X60 a X84 (violências aumiddlerovocadas), T50.9 a T65.9 (intoxicações exógenas de intenção indeterminada) e Y09 a X34 (acidente com intenção indeterminada). Não foram estabelecidos critérios de exclusão.

### Coleta de Dados

Por meio do Serviço de Arquivo Médico e Estatística (SAME) dos serviços estudados, foram localizados 420 prontuários de atendimentos relacionados aos códigos supracitados. Todos esses prontuários foram obtidos na íntegra e avaliados, sendo caracterizados tentativa de suicídio os casos em que havia documentação da intencionalidade, evidenciando, de maneira implícita ou explícita, o desejo do adolescente de tirar a própria vida^(6)^. Entre os prontuários analisados, 140 caracterizaram tentativas de suicídio e foram incluídos no estudo. A avaliação dos prontuários e a subsequente extração dos dados foram conduzidas por um enfermeiro, especialista em saúde da criança e do adolescente, com experiência clínica no atendimento de adolescentes que tentaram suicídio.

Para extração dos dados, foi utilizado um formulário elaborado pelos pesquisadores, composto por questões objetivas, com opções de respostas fechadas e abertas. Foram coletados dados referentes às características sociodemográficas dos adolescentes: sexo; cor (brancos e não brancos (pardos, negros, indígenas e amarelos)); idade; e escolaridade (até ensino fundamental (EF), ensino médio (EM) e ensino superior (ES)). Foram também coletados dados sobre diagnóstico prévio de transtornos mentais, uso atual de medicamentos psicotrópicos e fatores de risco comportamentais: tentativa de suicídio prévia; automutilação; ser LGBTQIA+; uso prévio e/ou atual substâncias psicoativas (SPA); histórico de abandono escolar e baixo desempenho escolar; fatores de saúde: relato de luto pelo falecimento de um ente querido; histórico de internação psiquiátrica prévia; alterações na percepção da imagem corporal; falta de acolhimento em tempo oportuno na Rede de Atenção Psicossocial (RAPS); e relato de vitimação por *bullying*; e os fatores de violência, conflito e estresse: histórico de vitimação por violência física, psicológica e sexual; relato de conflito familiar e amoroso; separação dos pais; e histórico de tentativa de suicídio por parte de um ente querido. Estas são as variáveis independentes. Reitera-se que os fatores de risco foram determinados a partir de estudos prévios^(10,12,14,15)^.

Para caracterização do diagnóstico prévio de transtornos mentais, considerou-se o registro de qualquer diagnóstico relacionado à seção F da CID-10. O uso atual de psicotrópicos foi considerado, nos casos em que havia registro de uso regular de algum medicamento psicotrópico, pertencente ao grupo N da classificação anatômica, terapêutica e química (ATC) da OMS nos 30 dias que antecederam o atendimento^(16)^.

### Análise e Tratamento dos Dados

Os dados foram tabulados e submetidos à análise descritiva e inferencial por meio do *software Stata18* ^®^ (*Stata Corporation, College Station*, Texas, USA). Para a análise descritiva, utilizaramse percentis, medidas de tendência central (média) e dispersão (desvio padrão), e para a análise inferencial, após teste da natureza de distribuição da variável, utilizou-se o teste qui-quadrado de Pearson para verificar a existência de associação entre as variáveis dependentes e independentes. Consideraram-se o alfa de 0,05 (ou 5%) e o Intervalo de Confiança a 95% para minimizar a ocorrência de erros tipo II.

### Aspectos Éticos

O estudo recebeu aprovação ética pelo Comitê de Ética e Pesquisa da Escola de Enfermagem da Universidade de São Paulo, sob Parecer n° 6.128.602, de 19 de junho de 2023, e da instituição coparticipante, sob Parecer n° 6.182.807, de 14 de julho de 2023. Respeitaram-se as diretrizes éticas estabelecidas nas Resoluções n° 466/12 e n° 510/16 do Conselho Nacional de Saúde. Houve dispensa da assinatura do Termo de Consentimento Livre e Esclarecido, com assinatura do Termo de Responsabilidade pela pesquisadora principal.

## RESULTADOS

Foram coletados dados de 140 prontuários de adolescentes atendidos por tentativa de suicídio. A idade média foi de 16 anos (DP: 2,2). Prevaleceram o sexo feminino (80,7%), brancos (80,7%) e que estudaram até o EM brasileiro (54,3%). Foi verificado diagnóstico prévio de transtornos mentais em 52,1% dos casos, com 36,4% dos adolescentes realizando uso de medicamentos psicotrópicos. Frente aos fatores de risco, destacou-se a alta prevalência de conflitos familiares (47,8%), tentativa de suicídio prévia (47,1%) e automutilação (30,7%) (Tabela 1).

**Tabela 1:** Caracterização dos adolescentes e fatores de risco (N* = 140) – São Paulo, SP, Brasil, 2023.

Caracterização dos participantes		
Variáveis	Média (+DP*)	Mín-Máx
**Idade (anos)**	16,3 (+2,2)	10–19
	N*	**Prevalência (%)**
**Sexo**		
Feminino	113	80,7%
Masculino	27	19,3%
**Cor**		
Branca	113	80,7%
Não branca	27	19,3%
**Idade**		
10 a 14 anos incompletos	35	25,0%
15 a 19 anos incompletos	105	75,0%
**Escolaridade**		
Até EF^‡^	44	31,4%
Até EM^§^	76	54,3%
Até ES^||^	20	14,3%
**Transtornos mentais**	73	52,1%
**Uso atual de medicamentos psicotrópicos**	51	36,4%
**Fatores de risco**		
	N*	**Prevalência (%)**
**Fatores de risco comportamentais**		
Tentativa de suicídio prévia	66	47,1%
Automutilação	43	30,7%
LGBTQIA^+¶^	13	9,3%
Uso prévio ou atual de SPA**	21	15,0%
Histórico de abandono escolar	4	2,8%
Baixo desempenho escolar	19	13,8%
**Fatores de risco de saúde**		
Luto	6	4,3%
Internação psiquiátrica prévia	2	1,4%
Alterações na percepção da imagem corporal	8	5,7%
Falta de acolhimento na RAPS^††^	1	0,7%
*Bullying*	15	10,7%
**Fatores de risco de violência, conflito e estresse**		
Violência física	5	3,8%
Violência psicológica	21	15,0%
Violência sexual	10	7,1%
Conflito familiar	67	47,8%
Conflito amoroso	24	17,1%
Separação dos pais	16	11,4%
TS^‡‡^ em alguém próximo	5	3,6%

Nota: *N = número de adolescentes;

^†^DP = desvio padrão;

^‡^EF = ensino fundamental;

^§^EM = ensino médio;

^||^ES = ensino superior;

^¶^LGBTQIA+ = lésbicas, *gays*, bissexuais, transexuais, *queers*, intersexuais, assexuais e outros;

**SPA = substâncias psicoativas;

^††^RAPS = Rede de Atenção Psicossocial;

^‡‡^TS = tentativa de suicídio.

A partir da análise inferencial, observou-se que a idade de 10–14 anos esteve associada à vitimação por *bullying* (p = 0,007), violência física (p = 0,004) e psicológica (p = 0,002), relato de conflito familiar (p < 0,001) e pais separados (p = 0,002). O sexo masculino associou-se ao uso de SPA (p = 0,01) e ao luto (p = 0,05). Ser adolescente não branco esteve associado ao histórico de abandono escolar (p < 0,001) e à violência sexual ­(p = 0,01). Os que estudaram até o EF apresentaram associação ao relato de conflitos familiares (p = 0,001) e à tentativa de suicídio por parte de um ente querido (p = 0,05). Já entre os adolescentes que estudaram até o EM, foi observada associação à falta de acolhimento na RAPS (p = 0,002). Diagnóstico prévio de transtornos mentais esteve associado à tentativa de suicídio prévia (p < 0,001), ser LGBTQIA+ (p = 0,01), alterações na percepção da imagem corporal (p = 0,005) e falta de acolhimento na RAPS (p = 0,002). Já o uso atual de medicações psicotrópicas associou-se ao histórico de tentativa de suicídio prévia (p = 0,005), ser LGBTQIA+ (p < 0,05) e presença de alterações na percepção da imagem corporal (p = 0,02). Nas Tabelas 2, 3 e 4, são apresentados os resultados dos testes de associação entre as variáveis dependentes e cada um dos fatores de risco incluídos no estudo.

**Tabela 2:** Associação entre as variáveis de caracterização e fatores de risco comportamentais (N* = 140) – São Paulo, SP, Brasil, 2023.

	TS^†^ prévia	p^‡^	Automutilação	p^‡^	LGBTQIA^+§^	p^‡^	Uso de SPA^||^	p^‡^	Abandono escolar	p^‡^	Baixo desempenho escolar	p^‡^
**Sexo**												
Feminino	52 (46,0%)	0,585	37 (32,7%)	0,287	9 (7,9%)	0,271	13 (11,5%)	**0,018**	2 (1,7%)	0,114	14 (12,4%)	0,403
Masculino	14 (51,8%)		6 (22,2%)		4 (14,8%)		8 (29,6%)		2 (7,4%)		5 (18,5%)	
**Cor**												
Branca	50 (44,2%)	0,160	36 (31,8%)	0,548	12 (10,6%)	0,266	14 (12,4%)	0,077	0 (0)	**<0,001**	17 (15,0%)	0,298
Não branca	16 (59,3%)		7 (25,9%)		1 (3,7%)		7 (25,9%)		4 (14,8%)		2 (7,4%)	
**Idade**												
10–14 anos	12 (34,3%)	0,078	14 (40,0%)	0,169	2 (5,7%)	0,401	4 (11,4%)	0,494	2 (5,7%)	0,241	5 (14,3%)	0,887
15–19 anos	54 (51,4%)		29 (27,2%)		11 (10,5%)		17 (16,2%)		2 (1,9%)		14 (13,3%)	
**Escolaridade**												
Até EF^¶^	15 (34,1%)	0,106	15 (35,1%)	0,839	3 (6,8%)	0,791	7 (15,9%)	0,795	2 (4,5%)	0,590	7 (15,9%)	0,160
Até EM**	41 (53,9%)		22 (28,9%)		8 (10,5%)		12 (15,8%)		2 (2,6%)		7 (9,2%)	
Até ES^††^	10 (50,0%)		6 (30,0%)		2 (10,0%)		2 (10,0%)		0 (0)		5 (25,0%)	
**Transtornos mentais**												
Sim	45 (61,6%)	**<0,001**	25 (34,2%)	0,344	11 (15,1%)	**0,014**	11 (15,1%)	0,981	3 (4,1%)	0,353	11 (15,1%)	0,589
Não	21 (31,3%)		18 (26,8%)		2 (2,9%)		10 (14,9%)		1 (1,5%)		8 (11,9%)	
**Medicação contínua**												
Sim	32 (62,7%)	**0,005**	20 (39,2%)	0,099	8 (15,7%)	**0,048**	7 (13,7%)	0,749	3 (5,8%)	0,104	9 (17,6%)	0,286
Não	34 (38,2%)		23 (25,8%)		5 (5,6%)		14 (15,7%)		1 (1,1%)		10 (11,2%)	

Nota: *N = número de adolescentes;

^†^TS = tentativa de suicídio;

^‡^p = valor de p;

^§^LGBTQIA+ = lésbicas, *gays*, bissexuais, transexuais, *queers*, intersexuais, assexuais e outros;

^||^SPA = substâncias psicoativas;

^¶^EF = ensino fundamental;

**EM = ensino médio;

^††^ES = ensino superior.

**Tabela 3:** Associação entre as variáveis de caracterização e fatores de risco de saúde (N* = 140) – São Paulo, SP, Brasil, 2023.

	Luto	p^†^	Internação psiquiátrica prévia	p^†^	Alteração na imagem corporal	p^†^	Falta de acolhimento	p^†^	Bullying	p^†^
**Sexo**										
Feminino	3 (2,6%)	**0,05**	1 (0,8%)	0,267	7 (6,2%)	0,616	1 (0,8%)	0,667	14(12,4%)	0,190
Masculino	3 (11,1%)		1 (3,7%)		1 (3,7%)		0 (0)		1 (3,7%)	
**Cor**										
Branca	5 (4,4%)	0,868	1 (0,8%)	0,267	7 (6,2%)	0,616	1 (0,8%)	0,667	14 (12,4%)	0,190
Não branca	1 (3,7%)		1 (3,7%)		1 (3,7%)		0 (0)		1 (3,7%)	
**Idade**										
10–14 anos	2 (5,7%)	0,630	0 (0)	0,411	3 (8,6%)	0,4	0 (0)	0,837	8 (22,8%)	**0,007**
15–19 anos	4 (3,8%)		2 (1,9%)		5 (4,7%)		1 (0,9%)		7 (6,8%)	
**Escolaridade**										
Até EF^‡^	3 (6,8%)	0,543	1 (2,3%)	0,771	1 (2,8%)	0,416	0 (0)	**0,002**	7 (15,9%)	0,162
Até EM^§^	2 (2,6%)		1 (1,3%)		5 (6,9%)		1 (1,3%)		8 (10,5%)	
Até ES^||^	1 (5,0%)		0 (0)		2 (10,0%)		0 (0)		0 (0)	
**Transtornos mentais**										
Sim	2 (2,7%)	0,346	1 (1,4%)	0,951	8 (10,9%)	**0,005**	1 (1,4%)	**0,002**	6 (8,2%)	0,319
Não	4 (5,9%)		1 (1,5%)		0 (0)		0 (0)		9 (13,4%)	
**Medicação contínua**										
Sim	1 (1,9%)	0,304	1 (1,9%)	0,688	6 (11,7%)	**0,02**	0 (0)	0,098	5 (9,8%)	0,792
Não	5 (5,6%)		1 (1,2%)		2 (2,2%)		1 (1,1%)		10 (11,2%)	

Nota: *N = número de adolescentes;

^†^p = valor de p;

^‡^EF = ensino fundamental;

^§^EM = ensino médio;

^||^ES = ensino superior.

**Tabela 4:** Associação entre as variáveis de caracterização e fatores de violência, conflito e estresse (N* = 140) – São Paulo, SP, Brasil, 2023.

	Violência física	p*	Violência psicológica	p*	Violência sexual	p*	Conflito familiar	p*	Conflito amoroso	p*	Pais separados	p*	TS^†^ próximo	p*
**Sexo**														
Feminino	4 (3,5%)	0,967	19 (16,8%)	0,219	7 (6,2%)	0,373	54 (47,8%)	0,973	19(16,8%)	0,833	14 (12,4%)	0,465	4 (3,5%)	0,967
Masculino	1 (3,7%)		2 (7,4%)		3 (11,1%)		13 (48,1%)		5 (18,5%)		2 (7,4%)		1 (3,7%)	
**Cor**														
Branca	4 (3,5%)	0,967	15 (13,3%)	0,242	5 (4,4%)	**0,011**	55 (48,7%)	0,693	20 (17,7%)	0,721	13 (11,5%)	0,954	4 (3,5%)	0,967
Não branca	1 (3,7%)		6 (22,2%)		5 (18,5%)		12 (44,4%)		4 (14,8%)		3 (11,1%)		1 (3,7%)	
**Idade**														
10–14 anos	4 (11,4%)	**0,004**	11 (31,4%)	**0,002**	4 (11,4%)	0,256	27 (77,1%)	**<0,001**	3 (8,6%)	0,12	9 (25,7%)	**0,002**	2 (5,7%)	0,430
15–19 anos	1 (0,9%)		10 (9,5%)		6 (5,7%)		40 (38,1%)		21 (20,0%)		7 (6,6%)		3 (2,8%)	
**Escolaridade**														
Até EF^‡^	3 (6,8%)	0,319	6 (13,6%)	0,085	4 (9,1%)	0,808	27 (61,3%)	**0,001**	8 (18,2%)	0,952	9 (20,4%)	0,071	4 (9,1%)	**0,05**
Até EM^§^	2 (2,6%)		15 (19,7%)		5 (6,6%)		38 (50,0%)		13 (17,1%)		6 (7,8%)		1 (1,3%)	
Até ES^||^	0 (0)		0 (0)		1 (5,0%)		2 (10,0%)		3 (15,0%)		1 (5,0%)		0 (0)	
**Transtornos mentais**														
Sim	2 (2,7%)	0,580	12 (16,4%)	0,619	5 (6,8%)	0,888	35 (47,9%)	0,983	10 (13,7%)	0,259	9 (12,3%)	0,727	3 (4,1%)	0,720
Não	3 (4,5%)		9 (13,4%)		5 (7,4%)		32 (47,7%)		14 (20,9%)		7 (10,4%)		2 (2,9%)	
**Medicação contínua**														
Sim	2 (3,9%)	0,866	9 (17,6%)	0,507	5 (9,8%)	0,355	24 (47,1%)	0,886	6 (11,7%)	0,201	8 (15,7%)	0,231	3 (5,8%)	0,265
Não	3 (3,4%)		12 (13,5%)		5 (5,6%)		43 (48,3%)		18 (20,2%)		8 (8,9%)		2 (2,2%)	

Nota: *N = número de adolescentes;

*p = valor de p;

^†^TS = tentativa de suicídio;

^‡^EF = ensino fundamental;

^§^EM = ensino médio;

^||^ES = ensino superior.

## DISCUSSÃO

A literatura^(6,10,17)^ indica que o final da adolescência é a etapa mais crítica do desenvolvimento, pela proximidade da transição à fase adulta, aumentando as cobranças, responsabilidades, expectativas familiares e sociais. Os adolescentes entre 15 e 19 anos tendem a tentar o suicídio quatro vezes mais do que os entre 10 e 14 anos^(6)^. Esse dado corrobora os achados deste estudo, com a prevalência de adolescentes entre 15 e 19 anos. Contudo, atenção especial deve se voltar a toda a fase da adolescência.

Neste estudo, 52,1% dos adolescentes tinham um transtorno mental, com associação a inúmeros fatores de risco. Esse achado corrobora a literatura^(6,9)^, que também indica que os transtornos mentais elevam em três vezes a possibilidade de tentativa de suicídio^(17)^. Há a necessidade emergente de um olhar à RAPS, com o avanço na tradução das políticas públicas à prática clínica, e o fortalecimento do serviço, especialmente nas suas ações de promoção, identificação precoce e acompanhamento, com ações de base comunitária, que se distanciam da visão hospitalocêntrica e biomédica, que guiaram a assistência em saúde mental por anos e que ainda influenciam a prática de cuidado infantojuvenil^(9)^. Entre as associações deste estudo, uma que merece atenção é a tentativa de suicídio prévia, que também se associou ao uso de medicações psicotrópicas contínuas.

A literatura indica que uma em cada quatro pessoas que se suicidam já tinha tentado suicídio, no mínimo, uma vez^(18)^. Isto corrobora uma coorte retrospectiva, com 25.037 jovens de 3 a 25 anos, atendidos em um serviço de saúde por demandas de saúde mental. Nesse, observou-se que 1.766 adolescentes tinham tentado suicídio nos últimos seis meses, sendo este fator o preditor mais forte para uma nova tentativa, com uma taxa de quatro vezes a mais^(19)^. Metanálise demonstrou que o risco de repetição está associado ao sexo feminino, por tentativas menos violentas, ao comparar com homens^(12)^, apesar de este estudo não demonstrar essa associação.

Em tentativas de suicídio recorrentes, a hospitalização tende a ser a conduta, todavia cabe refletir este contexto. Neste estudo, a internação psiquiátrica prévia não esteve associada a nenhuma variável dependente; já na literatura, observa-se que o suicídio é mais frequente nesse grupo, especialmente nos três meses após a alta^(18)^. No cenário brasileiro, após a consolidação da Reforma Psiquiátrica, o número de hospitalizações por problemas de saúde mental reduziu, com a transição de um cuidado hospitalocêntrico a um cuidado de base territorial, com destaque ao Centro de Atenção Psicossocial. Apesar da potencialidade desse aparato na RAPS, muitos profissionais ainda tendem a atuar com a medicalização da assistência em saúde mental, como visto neste estudo, porém é digno de nota que práticas hospitalocêntricas e de medicalização da assistência em saúde mental são questionadas na literatura, pois, isoladas, não promovem efeitos potenciais^(6,12,18)^.

No processo de cuidar em saúde mental, a família pode ser potencializadora da melhora, assim como pode promover novos desgastes. Isto é visto em estudo transversal com 22.126 adolescentes mexicanos, em que a presença dos pais em casa, bom relacionamento e clima agradável foram fatores de proteção à tentativa de suicídio. Já em casos em que o clima familiar foi classificado como ruim, a chance da tentativa de suicídio aumentou mais de duas vezes, e quando há um conflito, a chance aumentou três vezes^(17,19)^. Outra investigação transversal, realizada com 246 adolescentes na Colômbia, mostrou que 66% apresentaram algum tipo de disfunção familiar, sendo um preditor a transtornos mentais^(2)^. Esse achado corrobora este estudo, sendo o conflito familiar o fator de risco mais prevalente, podendo ser ocasionados por críticas, cobranças, quebra de expectativas e dificuldade de comunicação. Os conflitos podem ser ainda maiores em casos em que o adolescente abandona os estudos, como visto aqui, com associação entre conflitos e adolescentes que estudaram até o EF.

Nas situações adversas da adolescência, o luto por entes queridos pode ocorrer, como visto em 4,3% da amostra deste estudo, com associação ao sexo masculino, e isso corrobora revisão da literatura prévia, que demonstrou que o luto complicado pode levar ao risco do comportamento suicida e a outros transtornos mentais^(20)^. No caso de adolescentes do sexo masculino, isso pode ocorrer devido à sua representação social, construída desde a infância, em que a expressão de emoções deve ser reprimida para o encaixe em um padrão ideal.

Outra ocorrência é a tentativa de suicídio e/ou suicídio em familiares. A literatura indica que a prevalência de transtornos mentais está acima de 90% em familiares de indivíduos que se suicidaram^(13)^, podendo levar, também, ao comportamento suicida nos membros, especialmente os mais jovens^(21)^. Esse cenário pode ser traduzido a perda de entes queridos, como amigos. Aqui, esse fator esteve associado a adolescentes que estudaram até o EF. A relação familiar pode ser impactada por inúmeros fatores, o que leva ao adolescente buscar apoio e aceitação em outros contextos sociais, como o escolar.

Nas escolas, o *bullying* é frequente, como visto em revisão que indicou estimativas entre 18% e 31%. Ainda, este estudo demonstrou que a vivência do fenômeno leva ao isolamento social, desesperança, tristeza, falta de habilidades sociais e impulsividade. Outros impactos são baixo desempenho e posterior evasão escolar^(10)^, fatores vistos neste estudo. Para ressignificar essa vivência, é necessário que o adolescente adquira competências de enfrentamento, o que pode não ocorrer no início dessa nova fase da vida, hipótese para a associação entre o *bullying* e os adolescentes entre 10 e 14 anos, como visto neste estudo.

É digno de nota que parte dos fatores de risco pode ser, inicialmente, vivenciada nas escolas, o que demonstra a necessidade da articulação e fortalecimento dessa rede para o cuidado. No Brasil, em janeiro de 2024, foi aprovada a Política Nacional de Atenção Psicossocial nas Comunidades Escolares, que avança na atuação escolar, garantindo a promoção da saúde mental nesse local, com uma integração de diferentes serviços territoriais, garantindo a atenção psicossocial às crianças e adolescentes em risco^(22)^.

Aqui, a exposição a violências associou-se à idade e cor, com a violência psicológica sendo a mais prevalente. A literatura indica que a vitimação da violência leva a comportamentos de risco, como uso de SPA, alterações na percepção da imagem corporal, automutilação e demais transtornos mentais, sendo mais comum em menores que 16 anos^(23)^, corroborando este estudo que demonstrou associação entre a violência e adolescentes entre 10 e 14 anos. Investigação transversal, conduzida com jovens entre 13 e 24 anos, na Zâmbia (África), demonstrou que 76,8% foram vítimas de, no mínimo, uma violência; desses, 42,4% referiram sofrimento mental nos últimos 30 dias e, 12,5%, comportamento suicida^(24)^. Os dados são alarmantes, sendo necessário refletir sobre estratégias para dar visibilidade às violências, que são banalizadas dentro do senso comum e invisibilizadas na RAPS.

Outro fator de risco para tentativa de suicídio é fazer parte da comunidade LGBTQIA+, grupo exposto ao preconceito, por um conservadorismo histórico, que proporciona a exposição a inúmeros fatores de risco ao comportamento suicida. Inquérito populacional, realizado nos Estados Unidos, com 2.209 indivíduos LGBTQIA+, entre 12 e 29 anos que se suicidaram, demonstrou maior prevalência nos homens transexuais e mulheres bissexuais, maior probabilidade de tentativa de suicídio recorrente, transtornos mentais e uso de medicamentos^(25)^, semelhante a este estudo. Atenção especial deve se voltar a tentativas recorrentes, como visto em estudo em que, de 13.852 adolescentes LGBTQIA+, 276 tentaram suicídio uma vez, 116, entre 2-3 vezes, 37, entre 4–9 vezes, 19, acima de dez vezes, e 22, mais de dez vezes^(26)^.

No Brasil, estudo de base populacional, realizado com 510 adolescentes estudantes do EM, indicou a prevalência de risco de suicídio em 17,3%, número elevado, e esse risco foi 73% mais alto naqueles com alterações na percepção corporal^(7)^. As alterações na imagem corporal são um problema de saúde pública, todavia ainda silenciados, com enfoque na população adulta, quando suas repercussões se acentuam e levam às doenças crônicas e/ou risco iminente de vida. Na Coreia do Sul, estudo transversal demonstrou associação entre a alteração na imagem corporal, tanto obesidade quanto desnutrição, e a tentativa e/ou suicídio consumado^(27)^, o que corrobora este estudo, sendo este fator de risco aqui associado a problemas de saúde mental e uso contínuo de psicotrópicos.

Em meio a um sofrimento mental iminente, os adolescentes podem fazer uso de SPA como forma de se afastar da realidade. Em adultos, já é estabelecido que o uso de SPA pode aliviar sintomas indesejáveis, contudo, quando se torna a única forma de recreação, o risco de tentativas de suicídio se eleva^(11)^. Em adolescentes, o uso de SPA é um preditor ao comportamento suicida já estabelecido^(7,17)^, o que corrobora este estudo. Ainda, aqui o uso de SPA se associou ao sexo masculino, associação já estabelecida na literatura^(7,11,17)^.

Apesar de a automutilação não apresentar associação às variáveis deste estudo, sua prevalência foi alta (30,7%), corroborando a literatura^(14,18)^. Em metassíntese, observou-se que os jovens referiram se automutilar como forma de expressar suas emoções, com o alívio da dor emocional, compensada pela dor física^(14)^. Ainda, é digno de nota que esse comportamento, apesar de ser um fator de risco à tentativa de suicídio, pode não estar associado ao desejo de morrer, devendo ser investigado em um cuidado livre de estigmas^(18)^.

Ainda, um olhar a outros determinantes de saúde são necessários, como renda, moradia, segurança alimentar, apoio social, entre outros, que se interseccionam, potencializando os fatores de risco, mantendo ciclos de sofrimento emocional^(6,7)^. Neste contexto, a cor merece atenção, como os adolescentes negros, grupo que, historicamente, tem acesso desigual aos direitos sociais, e, ao acessarem, vivenciam opressões, potencializando vulnerabilidades^(28)^. Aqui, adolescentes não brancos tiveram associação ao abandono escolar e à violência sexual.

Neste estudo, apenas um adolescente tinha a documentação da ausência de acolhimento na RAPS. Apesar da baixa prevalência, sabe-se que este fenômeno é mais frequente. Alguns pontos podem estar associados à ausência de acolhimento oportuno como: o número reduzido de políticas públicas para a saúde de adolescentes, com as existentes focando na prevenção de uso de SPA, saúde sexual e reprodutiva; a perda de vinculação dos adolescentes com a RAPS, como na atenção primária, local onde as consultas de puericultura se voltam à primeira infância em detrimento das demais fases do desenvolvimento humano; e a busca prévia do serviço, com hostilização pelo profissional, em um atendimento marcado de estigmas, potencializando a visão de que o serviço não é um local potencial à sua vida^(3,4,7,14)^.

Há uma inexistência de espaços para o adolescente na contemporaneidade, sendo que atuar nos fatores de risco demanda, intrinsicamente, uma ressignificação desse contexto, com a redução da marginalização desse público perante a RAPS, que deve estar operando de forma interligada^(9)^. Avanço vem sendo construído nos últimos anos, com a Reforma Psiquiátrica brasileira, a implementação de serviços de base territorial e novas políticas de saúde intersetorial^(12,18,22)^. Ainda assim, um longo caminho ainda deve ser percorrido e, para tal, destacamos o papel essencial das pesquisas de implementação na tradução das políticas públicas à prática clínica, especialmente em locais em que seu acesso ainda não é garantido.

Ainda na organização do cuidado na RAPS, a atenção primária e a especializada merecem atenção. Nos adultos, estudo brasileiro demonstra que a interligação desses aparatos é quase que inexistente, e, em tentativas de articulação, nota-se uma perda na rede, com a responsabilização de apenas um serviço em detrimento dos demais^(29)^. Esta interligação pode ser mais desafiadora nos casos dos adolescentes, pelos aspectos supracitados nesta discussão.

Apesar de este estudo focar o olhar nos fatores de risco isolados, como demonstrado ao longo desta linha argumentativa, há uma somatória dos fatores que leva à potencialização das vulnerabilidades e sofrimento. Cabe refletir sobre estratégias para modificar esse cenário, com a atenção primária, por estar próxima aos adolescentes em seu território, sendo um aparato da RAPS essencial. Recomendam-se o estabelecimento de práticas de conscientização sobre saúde mental, lidando com o estigma, e a detecção precoce do comportamento suicida, com olhar aos fatores de risco interligados aos determinantes sociais, em uma perspectiva intersetorial, territorial e interdisciplinar, visando fortalecer os fatores de proteção^(6,10,18,30)^. Identificar os fatores de risco, aqui citados, e atuar diante de sua modificação, é o básico da linha de cuidado que visa modificar este cenário.

Neste estudo, houve a dependência da documentação dos profissionais de saúde sobre os fatores de risco e determinantes sociais, o que leva a uma limitação, uma vez que as estimativas de prevalência podem estar super ou subestimadas. Porém, os dados expostos são potenciais e inovadores à expansão da fronteira de conhecimento internacional sobre o comportamento suicida, contribuindo à enfermagem e à literatura.

Diante das contribuições do estudo à área da enfermagem, ressalta-se a possibilidade da qualificação da assistência a adolescentes na RAPS, com atenção àqueles que possuem fatores de risco ao comportamento suicida, permitindo intervenções rápidas e que possam reduzir a sua alta prevalência. Ressalta-se que a atuação da enfermagem diante do fenômeno não deve ocorrer apenas em serviços especializados em saúde mental, mas sim em toda a RAPS, sendo este estudo uma abertura para que os profissionais se aproximem e se empoderem dos fatores de risco, com conhecimento teórico que deve guiar ações na prática clínica. Já na literatura científica, indica-se a contribuição deste estudo com novas estimativas de prevalência dos fatores de risco em um cenário brasileiro, que devem ser trabalhados em estudos futuros que visem modificar este cenário, promovendo saúde mental e reduzindo a recidivas de tentativas de suicídio.

## CONCLUSÃO

Este estudo demonstrou a prevalência de fatores de risco comportamentais, de saúde, de violência, conflito e estresse, sendo os conflitos familiares (47,8%), tentativa de suicídio prévia (47,1%) e automutilação (30,7%) os mais prevalentes. Os fatores de risco se associaram à idade, sexo, cor, escolaridade, transtorno mental prévio e uso de medicação psicotrópica.
