# Supplementary material for: ERRATUM: Prevalence of risk factors among adolescents who suicide attempt: a cross-sectional study
Source: Rev Esc Enferm USP. 2025 Jan 27;58:e20240197er. doi: 10.1590/1980-220X-REEUSP-2024-0197eren (PMC11789624; doi:10.1590/1980-220X-REEUSP-2024-0197eren)
Supplement: Translation

# Translation: ERRATA: Prevalência de fatores de risco entre adolescentes que tentaram suicídio: estudo transversal

**DOI:** 10.1590/1980-220X-REEUSP-2024-0197erpt

No artigo “Prevalência de fatores de risco entre adolescentes que tentaram suicídio: estudo transversal”, com número de DOI: https://doi.org/10.1590/1980-220X-REEUSP-2024-0197pt, publicado na Revista da Escola de Enfermagem da USP, volume 58:e20240197 de 2024, p.1–10, na página 1:

**Onde se lia:**

Prevalence of risk factors among adolescents who suicide attempt: a cross-sectional study

**Leia-se:**

Prevalence of risk factors among adolescents who attempted suicide: a cross-sectional study
